# The Vagus Nerve Can Predict and Possibly Modulate Non-Communicable Chronic Diseases: Introducing a Neuroimmunological Paradigm to Public Health

**DOI:** 10.3390/jcm7100371

**Published:** 2018-10-19

**Authors:** Yori Gidron, Reginald Deschepper, Marijke De Couck, Julian F. Thayer, Brigitte Velkeniers

**Affiliations:** 1 SCALAB UMR CNRS 9193, Université Lille, BP 60149, Villeneuve d’Ascq CEDEX 59653, France; 2 Mental Health and Wellbeing Research Group, Vrije Universiteit Brussel (VUB), Laerbeeklaan 103, 1090 Jette, Brussels, Belgium; Reginald.Deschepper@vub.ac.be (R.D.); marijke.de.couck@vub.ac.be (M.D.C.); 3 Faculty of Health Care, University College Odisee, 9302 Aalst, Belgium; 4 Department of Neuroscience and Psychology, College of Medicine, Ohio State University, 33 Psychology Building, 1835 Neil Ave. Columbus, OH 43210, USA; thayer.39@osu.edu; 5 Faculty of Medicine and Pharmacy, Vrije Universiteit Brussel (VUB), Laerbeeklaan 103, 1090 Jette, Brussels, Belgium; brigitte.velkeniers@az.vub.ac.be

**Keywords:** global burden of diseases, neuroimmunology, neuromodulation, vagal nerve, prediction, prevention

## Abstract

Global burden of diseases (GBD) includes non-communicable conditions such as cardiovascular diseases, cancer and chronic obstructive pulmonary disease. These share important behavioral risk factors (e.g., smoking, diet) and pathophysiological contributing factors (oxidative stress, inflammation and excessive sympathetic activity). This article wishes to introduce to medicine and public health a new paradigm to predict, understand, prevent and possibly treat such diseases based on the science of neuro-immunology and specifically by focusing on vagal neuro-modulation. Vagal nerve activity is related to frontal brain activity which regulates unhealthy lifestyle behaviors. Epidemiologically, high vagal activity, indexed by greater heart rate variability (HRV), independently predicts reduced risk of GBD and better prognosis in GBD. Biologically, the vagus nerve inhibits oxidative stress, inflammation and sympathetic activity (and associated hypoxia). Finally, current non-invasive methods exist to activate this nerve for neuro-modulation, and have promising clinical effects. Indeed, preliminary evidence exists for the beneficial effects of vagal nerve activation in diabetes, stroke, myocardial infarction and possibly cancer. Thus, we propose to routinely implement measurement of HRV to predict such GBD in populations, and to test in randomized controlled trials effects of non-invasive vagal nerve activation on prevention and treatment of GBD, reflecting possible neuro-modulation of health.

## 1. The Problem

Major non-communicable causes of death and of years of life lost today include coronary heart disease (CHD), stroke, cancer and pulmonary diseases [1]. Many risk factors (pollution, smoking, diet-driven cholesterol, insufficient exercise, etc.) explain a large proportion of major global burden of diseases—GBD (e.g., [2]). Furthermore, many of these diseases have common underlying biological causes, as we shall see below.

While modernization has brought many positive developments (e.g., transportation, huge improvements in disease detection and treatment, immense improvements in access to information via IT), it has heavy environmental, occupational and lifestyle consequences, with vast health implications. For example, modernization leads to more air pollution which is expected to double by 2050 [3], to a more sedentary lifestyle with reduced physical activity, and more job stress, all having immense and adverse health consequences [4]. Indeed, worryingly increasing trends have been observed in the prevalence of elevated body mass index (BMI) and in diabetes in several world regions [5]. Concerning ischemic heart disease (IHD), while rates of myocardial infarction and angina pectoris have decreased from 1990 to 2010, the disability-adjusted life years (IHD-burden) increased by 29% in that period [6]. Thus, better treatment saves lives and may prevent the diseases, but among people with CHD, disability is higher. In cancer, an increase in colon cancers has been observed in several countries [7] while incidence of breast cancer has seen increases followed by decreases in western countries [8]. Importantly, while there is a global decrease in disability-adjusted life years (DALY) from communicable, maternal, neonatal and nutrition-related diseases, there has been an increase in DALY from non-communicable diseases between 1990 and 2016 [9]. Another trend is the expected increasing burden from chronic obstructive pulmonary disease (COPD), due to increasing pollution in some world regions and due to the aging population [10]. Aiming to fight these trends, is there one resilience factor which is epidemiologically related to major causes of death, which is related to behavioral risk factors contributing to these diseases, and which is related to their common underlying pathophysiological causes? If such a factor were found, harnessing it would be of immense importance for global public health. We hereby present the vagus nerve hypothesis for global public health, reflecting a neuro-immunological paradigm. The potential modulatory role of the vagus nerve has been previously related to some chronic diseases [11,12]. In this article, we shall focus on three pathophysiological causes of many major health problems, namely oxidative stress, inflammation and excessive sympathetic activity. We then bring scientific evidence for the association between these causes of major diseases and vagal nerve activity and epidemiological evidence showing that higher vagal activity predicts reduced risk of major chronic diseases. Finally, we provide past and recent evidence for the effects of vagal nerve activation on GBD-related outcomes, and end with a note of caution.

## 2. Oxidative Stress and Chronic Diseases

Oxidation is the process whereby important molecules lack electrons in their atoms. Oxidative stress occurs when the body has more pro-oxidants than anti-oxidants, crucial if megamolecules such as DNA become damaged. Under some conditions of hypoxia (see below), oxidative stress may also develop. A major adverse effect of modernization is pollution, which has dramatically increased over the past centuries. Pollution-induced oxidative stress is linked to cardiovascular diseases and cardiac death [13]. Pollution also causes inflammation to induce oxidative stress and subsequent DNA damage, obvious contributing factors in carcinogenesis [14]. Air pollution is also a risk factor for dementia [15], of increasing relevance due to the global aging population. Looking at psychological stress, job stress is also associated with higher oxidative stress (hydrogen peroxide; [16]). Job stress may increase in the next decades due to more globalization, competition and rapidly changing job schemas and uncertain job security. Furthermore, chronic stress was found to be related to reduced tellomere length [17], which predicts diseases and earlier mortality (e.g., [18]). More specifically related to oxidative stress, higher effort-reward imbalance at work, indicating chronic work stress, was associated with higher levels of hydrogen peroxide, in Japanese men [16].

## 3. Inflammation and Chronic Diseases

Inflammation reflects recruitment of immune cells (mostly innate) due to various danger signals (e.g., injury, infection, cell damage). Inflammation contributes to all stages of carcinogenesis from escape from apoptosis and tumor onset [19], to angiogenesis and metastasis [20,21]. Inflammation contributes to multiple stages of atherogenesis via plaque formation by macrophage recruitment [22] to the acute coronary syndrome via creating plaque instability by macrophages, plaque rupture and superimposed thrombosis [22,23]. In a meta-analysis on COPD, multiple inflammatory markers were associated with COPD [24]. Inflammation is also a major contributing factor to insulin resistance [25], the main factor underlying diabetes. Finally, psychosocial stress is related to elevated pro-inflammatory cytokines and to reduced anti-inflammatory cytokines, in vulnerable individuals [26]. Thus, inflammation, the over-reaction of the immune system to danger signals, underlies multiple non-communicable chronic diseases.

## 4. Excessive Sympathetic Nervous System Activity and Chronic Diseases

Excessive sympathetic nervous system (SNS) activity is related to cardiovascular diseases [27] by inducing greater oxygen demand from the heart and by inducing vasoconstriction, which can induce ischemia. High SNS activity also affects the direction to which cancer cells will metastasize [28]. Furthermore, sympathetic activity, indexed by muscle sympathetic nerve activity, is high in COPD and predicts poor prognosis in COPD [29]. In addition, excessive sympathetic activity was found in diabetic patients to predict increased risk of cerebral and cardiovascular events [30]. Diabetes is a major global concern of public health due to its increasing prevalence and because it is a risk factor of CHD and certain cancers on its own [31]. Finally, job stress is associated with higher SNS activity as well. Globalization and increases in economic competition could result in future higher job stress, reduced work security, and subsequently in more burnout [32]. In a study on job stress in bus drivers, driving in peak traffic was associated with elevated catecholamines [33], sympathetic neuro-hormones. This is important given the constant global increase in traffic congestions with increasing wealth [34]. Thus, the prevalence and possible impact of increased SNS activity on health are expected to rise in the future.

## 5. Introducing Neuro-Immunology and Neuro-Modulation to Public Health

Medical practice often seeks to treat each trigger or contributing factor of diseases separately, by the best available method. However clinically and economically, finding one factor which can inhibit all three aforementioned factors at once (oxidative stress, inflammation and sympathetic hyperactivity), would be far more efficient, and may result in fewer side effects than by three separate medications. Furthermore, if such an inhibiting factor would be found, measuring its activity should also predict reduced disease risk. These prognostic and therapeutic issues have crucial relevance to public health. We propose here that the vagus nerve (the wandering nerve) may fulfill all these criteria, as detailed below. This knowledge stems from the domain of neuro-immunology, the science which investigates the interplay between the nervous and immune systems, which is more relevant to health than previously thought so. The vagus nerve is the 10th cranial nerve, descending from the brain stem and arriving to most visceral organs. The vagus nerve is a major branch of the parasympathetic nervous system, among other nervous such as the facial and glossopharyngael nerves. Vagal nerve activity is non-invasively indexed by heart-rate variability (HRV), the fluctuations in normal R-R heart beat intervals. Actual vagal nerve activity is very strongly correlated with HRV (r = 0.88) and vagomimetic medications have profound effects on HRV [35]. Certain specific indexes of HRV, namely route mean square of successive differences (RMSSD) and the high-frequency domain HRV (HF-HRV) reflect pure vagal control of heart rate oscillations. High HRV is also related to shorter stress responses in hormonal, cardiac and immune (inflammatory) markers [36], demonstrating the homeostatic role this nerve plays. Furthermore, in people exposed to stress, only in those with high HRV, brain activity was found to be synchronized to and was correlated with peripheral immune and hormonal stress responses [37]. This demonstrates the role of the vagus in bridging and in synchronizing brain and peripheral systems, key for neuro-modulation. Importantly, vagal nerve activity predicts the risk of and prognosis in many major GBD, as we now shall see.

## 6. Epidemiological Path: Vagal Nerve Activity Predicts Risk and prognosis of Chronic Diseases 

Epidemiological evidence shows inverse associations between vagal nerve activity, indexed by HRV, and the full metabolic syndrome as well as with the number of its components [38]. At the population level, high HRV predicted reduced risk of overall mortality and reduced risk of cancer death [39]. A meta-analysis of 21 studies found that myocardial infarction patients with the SDNN HRV index below 70 ms later had approximately four times the risk of mortality, compared to those with SDNN > 70 ms [40]. A more recent meta-analysis shows that higher HRV significantly predicts longer survival in cancer [41], and such an association was found to be statistically mediated by reduced inflammation, specifically in pancreatic cancer [42]. Concerning diabetes, HRV is inversely related to insulin resistance [43] and to levels of HbA1C [44]. Finally, HRV is also associated with complications in COPD [45]. Thus, HRV, the vagal nerve index, could be used to predict onset and prognosis of major global disease burdens. Its ease of measurement (via ECG or finger pulse devices) and its independent prognostic role, call for physicians and health policy makers to implement using this biomarker routinely for prediction and possible prevention of major diseases in the population.

## 7. Biological Path: The Vagus Nerve Inhibits Oxidative Stress, Inflammation and Sympathetic Activity

Empirical evidence exists to support the contention that the vagus nerve inhibits all three major disease-promoting biological factors mentioned above. First, vagus nerve stimulation (VNS) reduces oxidative stress [46]. More recently, Bezerra et al. [47] found in mice with a myocardial infarction (MI), that VNS reduced protein oxidation. Second, the vagus plays a key neuro-immune mediator and modulator between peripheral immune signals and the brain. The vagus informs the brain about low-level peripheral inflammation via its receptors for the inflammatory cytokine interleukin-1 [48]. Importantly, the vagus then inhibits inflammation via two routes. The first one is via activation of the hypothalamic pituitary adrenal axis which results in systemic cortisol secretion which reduces inflammation [49]. The second route is via vagal and then sympathetic branches arriving at the spleen, reflecting cholinergic and then noreadreneric signals that trigger certain splenic T-cells via adrenergic receptors. These T-cells then secrete the vagal neurotransmitter acetylcholine which binds to the alpha-7 nicotinic acetylcholine receptor on monocyte, resulting in the inhibition of synthesis of inflammatory cytokines [50]. Together, these two routes constitute the vagal anti-inflammatory reflex. Third, activity of the vagus nerve, being a major part of the parasympathetic nerve system, inhibits sympathetic activity [51]. An important parasympathetic role of the vagus is vasodilation. This is done specifically by vagal nerve induced increases in vasoactive intestinal peptide, which then increases coronary blood flow [52]. This anti-hypoxic role is crucial for reducing risk of CHD, stroke and even also cancer since many tumors flourish in hypoxic conditions and hypoxia is prognostic in cancer [53]. Interestingly, there is evidence that hypoxia (related to excessive sympathetic vasoconstrictive activity), oxidative stress and inflammation are causally related in a vicious circle manner [54]. Thus, biologically, the vagus nerve inhibits all three promoters of the major chronic diseases mentioned above, and empirical evidence supports this.

## 8. Behavioral Path: Effects of Vagal Activity on Lifestyle Risk Factors of Chronic Diseases

As mentioned above, many lifestyle and behavioral risk factors (smoking, diet-driven cholesterol, insufficient exercise, etc.) predict or explain a large proportion of GBD (e.g., [2]). Vagal nerve activity is associated with these lifestyle risk factors in a bi-directional manner. For example, HRV was significantly lower in smokers than non-smokers, and HRV decreased after smoking among smokers [55]. Importantly, vagus nerve activity, indexed by HRV, is positively related to executive functioning [56]. Executive functioning is an overarching neurocognitive construct which includes self-regulation, inhibition, memory and problem-solving. Executive functioning is inversely relate to and can modulate risk factors such as smoking, unhealthy diets and sedentary behaviors [57]. Furthermore, high executive functioning increases the association between intentions to adopt healthy behaviors and their actual adoption [58]. Thus, potentially, by increasing vagus nerve activity, behavioral risk factors of diseases may be diminished as well, via increased executive functioning. Furthermore, transcutaneous vagus nerve stimulation (tVNS) led to reduced activity in limbic brain regions [59], and recently also to increases in the anterior cingulate and the left prefrontal cortex [60]. Together, this pattern may suggest that tVNS increases executive control and emotional regulation, possibly relevant to ‘emotional eating’ and to greater frontal control of GBD behavioral risk factors in general.

Indeed, empirical evidence supports such claims. Vagal nerve stimulation led obese rats to consume less food, and subsequently, to lose weight [61]. One study found that people who were high on food craving, those performing HRV-biofeedback, a method of self-activating one’s vagal nerve (via HRV), subsequently reported reductions in food craving compared to controls [62]. Finally, one study linked HRV to both executing functioning brain regions and self-control concerning eating. Higher HRV was associated with better self-control of craving for certain foods and with higher brain activity in the ventromedial prefrontal cortex, a region crucial in decision-making [63]. As mentioned above, it is important to note that some of these associations are bi-directional—for example, physical activity increases HRV [64].

## 9. Implications for Prevention and Treatment: Activating the Vagal Nerve for Health

Several non-pharmacological methods exist for activating the vagus nerve. First, electric invasive and now also transcutaneous and non-invasive vagus nerve stimulation (nVNS) devices have been developed. Invasive VNS reduced food and weight in rats [61]. nVNS devices were found to reduce chronic headaches [65], to reduce inflammation [66] as well as depression [67]. Another study found that nVNS led to reductions of glucose and HbA1C levels in diabetic rats [68]. The second non-invasive manner is a self-activating form achieved by performing paced vagal breathing while receiving feedback on one’s HRV, called HRV-biofeedback (HRV-B). One study found HRV-B to induce changes in inflammation in hypertensive patients as a function of change in HRV [69]. Furthermore, in a matched case-control pilot study on *n* = 6 patients with metastatic colon cancer, De Couck et al. [70] found that after 3 months of HRV-biofeedback + chemotherapy, strong reductions in the tumor marker carcino-embryonic antigen (CEA) were seen, compared to well-matched controls receiving chemotherapy alone. Yet, such a small study without randomization must be replicated. Various forms of meditation and yoga also increase HRV [71].

There are several pharmacological manners to activate the vagal nerve, but we will focus on one. Semapimod is a vagal-dependent anti-inflammatory drug. It was found to increase sensitivity of microliga to radiotherapy, in an animal model of glioblastoma [72]. This could have profound implications for treating this very aggressive brain tumor.

One recently developed method for activating the vagus electrically is intravenous VNS (iVNS), which was recently found to reduce infarct size in an animal model of myocardial infarction [73]. In that study, compared to control dogs whose relative infarct size was 13.3%, dogs receiving iVNS immediately post-infarct had a 2.4% infarct size, and those receiving it 90min later had a 4.5% infarct size. In another study, animals induced to have a stroke, showed faster recovery of motor skills when given VNS that was paired with the training [74]. In an animal model of obesity-related diabetes, VNS led to increased whole-body glucose uptake and to increased insulin sensitivity and reduced fasting glucose [75]. This could have profound implications for diabetes, a risk factor of many of the GBD mentioned here. In the coming years, studies need to test effects of such vagal nerve activating interventions on the chronic diseases mentioned above, using RCTs.

## 10. Note of Caution

Despite the epidemiological, behavioral and biological evidence for the rationale to monitor and possibly activate the vagal nerve in multiple chronic diseases, some contrary evidence exists. First, in cancer, non-neuronal autocrine/paracrine acetylcholine, the major vagal nerve neurotransmitter, may in fact potentiate cell growth, because inhibiting its precursor by Bromoachetylcholine bromine, reduced colon cancer cell proliferation [76]. Furthermore, similar results were also found in lung cancer [77]. It is possible that autocrine non-neuronal acetylcholine in tumor cells promotes carcinogenesis while systemic vagal nerve effects may reduce cancer progression. In addition, over-activation of the vagal nerve may induce syncope and asystole [78]. Thus, the differences between systemic and local effects of the vagal nerve neurotransmitter require further investigation, and the activation of this nerve needs to be done within a safety range.

From a methodological point of view, issues of 3rd variables need to be considered thoroughly when conducting longitudinal observational or interventional RCT, to isolate the hypothesized contribution of the vagus nerve in preventing GBD. In observational studies, it is crucial to measure HRV for at least 5 min, to obtain both time and frequency domain HRV parameters in a reliable manner. It is crucial to assess and statistically adjust for the effects of lifestyle factors such as smoking, physical activity and diet, all which are related to HRV (e.g., [79]). In interventional studies (RCT), it is important to considering matching patients on 3rd variables, as was done in other domains [80], to methodologically rule our their effects. Finally, if vagal nerve activation will be tested and used in the future to possibly prevent and treat certain GBD, issues of frequency, duration and intensity of vagal activation need to be tested per disease, while always considering safety issues.

## 11. Summary

**Figure 1:**
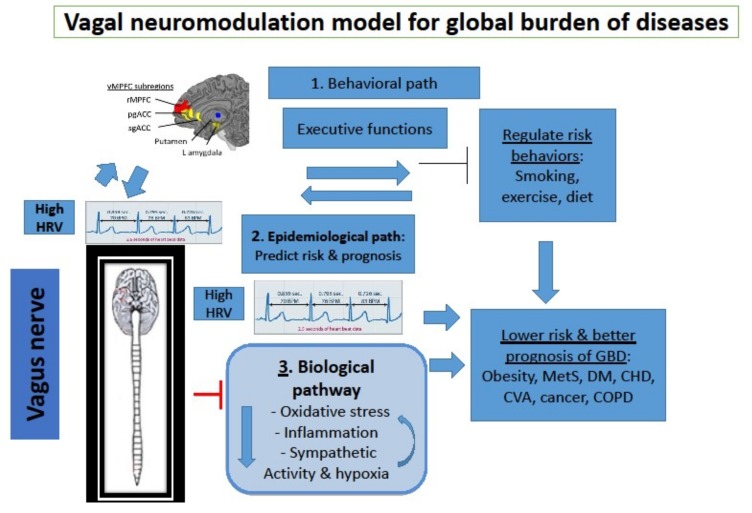
The relationship between vagal nerve activity and global burden of diseases, via three pathways: Behavioral, epidemiological and biological.

Chronic non-communicable diseases can be fatal, they are prevalent and pose a huge individual, social, medical and economic burden. However, they are linked to several common lifestyle (behavioral) and pathophysiological factors contributing to their onset. The present article introduces a novel paradigm and approach to such prevalent public health problems, namely a neuro-modulation approach, based on the science of neuro-immunology. Figure 1 depicts our proposed role of the vagal nerve in prediction and possible prevention of major diseases, via three paths: Behavioral, epidemiological and biological. The vagus nerve reduces certain behavioral risk factors and inhibits three main pathophysiological factors which contribute to those diseases (oxidative stress, inflammation and excessive SNS activity). This may cut a vicious circle existing among these three pathophysiological factors. At the epidemiological level, HRV, the vagal nerve index, predicts the risk of these diseases, and several studies show beneficial effects of vagal nerve activation on the pathophysiological contributors, on the behavioral risk factors and on some of these fatal diseases. It is recommended to use HRV to estimate risk of major GBD in various populations. Future studies should test in large-scale trials the effects of vagal nerve activation on these diseases, to reduce the global burden resulting from them. If effective, such a paradigm may reveal new mechanisms for understanding these diseases, and a more economic and possibly more efficient manner for detecting and treating large segments of the population who are at risk for chronic diseases via neuro-modulation, by adopting neuro-immunology in public health.
